# Mobile App Use by Primary Care Patients to Manage Their Depressive Symptoms: Qualitative Study

**DOI:** 10.2196/10035

**Published:** 2018-09-27

**Authors:** Alison Pung, Susan Louise Fletcher, Jane Maree Gunn

**Affiliations:** ^1^ Department of General Practice The University of Melbourne Victoria Australia

**Keywords:** mobile apps, depression, health care, general practice

## Abstract

**Background:**

Mobile apps are emerging as tools with the potential to revolutionize the treatment of mental health conditions such as depression. At the forefront of the community health sector, general practitioners are in a unique position to guide the integration of technology and depression management; however, little is currently known about how primary care patients with depressive symptoms are currently using apps.

**Objective:**

The objective of our study was to explore the natural patterns of mobile app use among patients with depressive symptoms to facilitate the understanding of the potential role for mobile apps in managing depressive symptoms in the community.

**Methods:**

Semistructured phone interviews were conducted with primary care patients in Victoria, Australia, who reported symptoms of depression and were enrolled in a larger randomized controlled trial of depression care. Interviews explored current depression management strategies and the use of mobile apps (if any). Interviews were audio-recorded and transcribed verbatim. Inductive thematic analysis was iteratively conducted using QSR NVivo 11 Pro to identify emergent themes.

**Results:**

A total of 16 participants, aged between 20 to 58 years, took part in the interviews with 11 reporting the use of at least one mobile app to manage depressive symptoms and 5 reporting no app use. A variety of apps were described including relaxation, mindfulness, cognitive, exercise, gaming, social media, and well-being apps to aid with depressive symptoms. Among users, there were the following 4 main patterns of app use: skill acquisition, social connectedness, inquisitive trial, and safety netting. Factors that influenced app use included accessibility, perceptions of technology, and personal compatibility. Health care providers also had a role in initiating app use.

**Conclusions:**

Mobile apps are being utilized for self-management of depressive symptoms by primary care patients. This study provided insight into the natural patterns and perspectives of app use, which enhanced the understanding of how this technology may be integrated into the toolbox for the management of depression.

**Trial Registration:**

Australian New Zealand Clinical Trials Registry ACTRN12616000537459; https://www.anzctr.org.au/Trial/Registration/TrialReview.aspx?id=367152 (Archived at WebCite at http://www.webcitation.org/71Vf06X2T)

## Introduction

Depression is a globally prevalent and costly condition affecting more than 300 million people worldwide [1]. In Australia, 1 in 7 women and 1 in 12 men experience a depressive episode at some stage of their lives [2]. Although the vast majority of cases are mild to moderate in severity [2], the economic burden of depression on the community is high due to direct medical costs and loss of productivity as the rate of service utilization continues to climb [3,4]. It is a leading cause of disability with significant impact on morbidity and individual functioning; however, only 35% of people with depression will access treatment [2].

For those who do seek help, general practitioners are commonly the first point of contact; three quarters of people receiving any mental health care reported seeing a general practitioner [5,6]. Consequently, general practitioners are in a unique position to optimize the detection and treatment of depression. However, studies show that issues such as time constraints, affordability, and lack of access to mental health specialists are common barriers to managing depression in primary care [7].

As ubiquitous devices that are owned by 79% of the population [8], mobile phones have become increasingly integrated into daily lives and may represent a novel solution to overcoming these barriers in primary care depression management. One avenue by which mobile phones may improve the delivery of mental health interventions is through mobile app software specifically designed for use on mobile phones. Apps are advanced technological tools with multiple capabilities and have been postulated to revolutionize mental health treatment in myriad ways, such as by allowing for the affordable and accessible delivery of interventions, providing real-time diagnostic and monitoring support, enhancing therapeutic relationships, augmenting engagement with treatments, and even acting as “virtual coaches” [9-11]. Consequently, interest in harnessing the potential of mobile apps for the management of depression is mounting from patients, clinicians, and the broader community [12,13].

In recent years, a vast number of mobile apps have been developed to address the diverse aspects of depression management including screening, symptom tracking, psychoeducation, stress management, medication support, and cognitive behavioral therapy [14,15]. Researchers have sought to make sense of this expanding environment by analyzing the app marketplace, reviewing the scientific literature, and conducting trials of specific apps [14-18]. Although emerging evidence supports the efficacy of apps in reducing the severity of depressive symptoms, a recent meta-analysis identified only 22 randomized controlled trial-tested apps [16], whereas a content analysis of the app marketplace in 2015 revealed 243 clinically relevant depression apps available for download [14]. Therefore, the vast majority of apps available have not been vigorously tested, and establishing an evidence base for mental health apps remains an ongoing challenge because the rate of app development far outstrips that of research [17-20].

The challenge of navigating this new health care sector alone should not be underestimated and in their role at the forefront of depression treatment in the community, general practitioners can play a vital part in connecting technology and traditional mental health care [21]. A pilot trial of a mobile app in a primary care setting demonstrated that a mobile platform can be acceptably integrated into the therapeutic relationship between care providers and patients to augment depression management [22]. However, to optimize integration, it is important to first understand the baseline app use, and there remains a limited literature on this topic. In 2014, Bauer et al [23] reported that only a minority of primary care patients (22%) used a health app. In contrast, a 2017 US survey of people with symptoms of anxiety or depression demonstrated that 78% people had a health app installed on their phone and typically used each app for a single purpose (eg, symptom tracking, habit building, or providing a routine) [24]. It is worth noting that this study involved participants who were screened for enrollment in a trial of the IntelliCare suite of mental health apps, which could account for the high numbers of IntelliCare apps reported. In addition, both these studies assessed only general health app use and not the extent to which apps were used specifically to manage mental health.

At the same time, most app research in the mental health space has focused exclusively on mental health-specific apps; hence, the utility of other app types for depression management is also uncertain. To harness the potential of mobile apps and enable their effective integration in primary care, understanding how patients naturally use apps to manage depressive symptoms is critical. Hence, the primary aim of this study was to conduct an in-depth qualitative examination of common mobile app usage patterns for depression in the primary care population to gain a better understanding of how general practitioners may best integrate apps into their routine depression care.

## Methods

### Study Design, Setting and Recruitment

To explore the natural patterns of mobile app use for depressive symptoms among primary care patients, this study employed a qualitative design using individual semistructured phone interviews. Ethics approval for this study was granted by the Human Research Ethics Committee of The University of Melbourne (ID: 1543648.9).

Participants were recruited from the Target-D trial (ACTRN12616000537459). This is a large Australian study of a clinical prediction tool to triage and target depression treatment, in which patients with depressive symptoms were directly recruited from primary care practices across metropolitan Melbourne, Victoria [25]. On completion of baseline data collection, participants were stratified into mild, moderate, and severe symptom groups and randomly allocated to receive usual care or a symptom severity-matched treatment as follows: internet-based self-help, guided internet-based cognitive behavioral therapy, or nurse-led collaborative care (for the mild, moderate, and severe groups, respectively).

Participants were eligible for the Target-D trial if they were aged 18-65 years, had current depressive symptoms (assessed during the screening process as scoring 2 or more on the two item version of the Patient Health Questionnaire, PHQ-2), had access to the internet, and had sufficient English language comprehension to provide informed consent. Participants were excluded if they were taking an antipsychotic, had recently started or changed their antidepressant, were currently receiving psychological treatment, or were currently using an internet-based cognitive behavioral therapy program.

The only additional eligibility criterion applied to this study was that participants had completed the 3-month intervention phase of a randomized controlled trial to avoid prompting those in the usual care group to change their approach to mental health care.

Purposive sampling was undertaken from randomly generated lists of Target-D participants, 30 at a time, stratified by depressive symptom severity assessed using the PHQ-9 [26] to ensure a mix of age and gender in the final sample. The interviewer was blinded to whether participants were allocated to the control or intervention groups.

**Figure 1:**
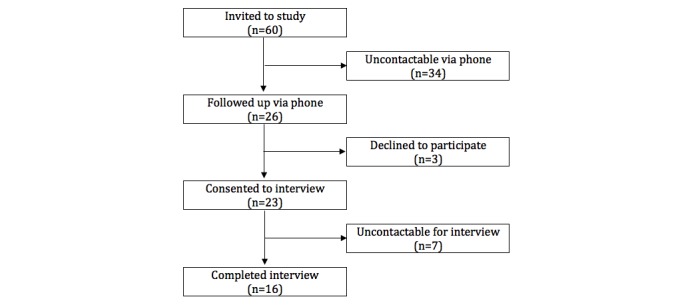
Participant flow.

Potential participants were sent an email invitation to take part in the study and followed up via phone call within 2 weeks to establish interest, obtain informed consent, and arrange a suitable interview time. Of the 60 participants who were emailed invitations, 26 were contactable by phone and 16 took part in the study, as seen in Figure 1. Of those who did not participate, 3 did not consent (one did not provide a reason, one was in hospital, and one was uninterested in the study) and 7 initially consented to the interview; however, they were lost to follow-up thereafter.

### Data Collection

Semistructured phone interviews were conducted with 16 participants between May and October 2017 by a female general practice registrar who had no prior contact with the participants. This was conducted using an iterative approach, where data collection occurred incrementally and simultaneously alongside data analysis.

Interviews began by asking participants about their current depression management strategies to establish if mobile apps were used. If any mobile apps were mentioned, this was explored in further detail including type, purpose, duration, referral source (ie, how participants became aware of the app), perceived challenges, benefits, and recommendations. If no apps were mentioned, questions focused on attitudes to using mobile apps for managing depressive symptoms. The semistructured interview discussion guide can be viewed in Multimedia Appendix 1.

All participants consented to audio recording of the interviews, which ranged from 10 to 32 minutes. Recordings were subsequently transcribed verbatim by an independent professional transcription service and verified by AP. Field notes were created during and after the interviews and data were stored confidentially on protected university servers. Participants were offered an Aus $20 gift voucher as an honorarium.

### Data Analysis

To identify the natural patterns of app use, inductive analysis was employed utilizing NVivo 11 Pro software (QSR International, Melbourne, Australia) to organize and identify core themes and subthemes in accordance with Braun and Clarke’s 6 phases of thematic analysis [27]. This consisted of several stages including familiarization, transcription, generating initial codes, searching for themes, reviewing themes, and then defining and deciding on meaningful themes.

An iterative approach was adopted with the initial coding performed by a general practice registrar following the completion of the first set of 5 interviews. Subsequent interviews were then conducted incrementally with data analysis occurring simultaneously, and new codes were added from the dataset to NVivo Pro 11. SF and JG reviewed the transcripts and codes during this process until consensus was reached by all members of the research team in regards to the final themes. Data saturation was achieved (and data collection ceased) when no new themes emerged, which was determined by the point in analysis where no new codes were able to be created in NVivo 11 Pro that provided additional value to the identified themes [28,29].

## Results

### Participant Characteristics

The age of the participants ranged from 20 to 58 years. Overall, 58% (9/16) were female and the majority had achieved at least a tertiary level of education. Three quarters of participants had PHQ-9 scores indicating moderate to moderately severe depression. On average, participants completed the interview 269 days after receiving their Target-D treatment recommendation (range 99-369 days). Further demographic information is provided in Table 1, whereas Table 2 details app use and participation in Target-D, including whether Web-based tools were recommended.

### Patterns of App Use

**Figure 2:**
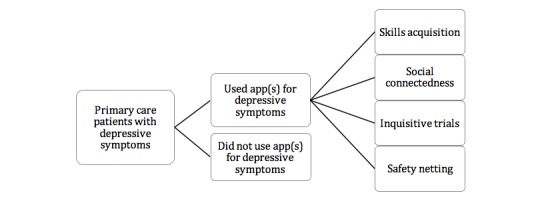
Patterns of app use.

Overall, 11 out of 16 participants reported using at least one mobile app to manage their depression with 15 apps mentioned in total (Table 3). All apps were free to download and included, but were not limited to, mental health-specific apps. Awareness of the apps came from numerous sources including self-directed research, inbuilt on phone, and recommendations from friends, organizations, and health professionals. Participants could be divided into 2 groups, users and nonusers. Among users, the following 4 main patterns of app use were identified: skill acquisition, social connectedness, inquisitive trial, and safety netting, as seen in Figure 2.

#### Skill Acquisition

Three participants used their mobile apps on a regular basis over a short period, generally several months, to learn, reinforce, or master an activity that they deemed helpful for their mental health. Participants tended to hold a biopsychosocial perspective of their treatment needs with a focus on self-help management strategies with known efficacy for depression such as exercise, mindfulness, and social connectedness. Apps were often sought and utilized to support these perceived needs and enhance preferred strategies.

Monitoring or tracking progress via an app was one method that aided in reinforcing an activity that a participant deemed useful for improving mood.

I was using exercise apps and that, like tracing my steps...I mean I found it quite useful, I guess in sort of being more mindful in movement...I found that kind of focused me for a while

P2

An app could also be used to learn and master a skill. One participant, who reported suffering from severe anxiety, used a mindfulness app daily for 6 months to master the exercises which ultimately enabled her to maintain control of her symptoms.

...then I got to the point that I was able to just sit there and get into my own headspace and just do the meditation and breathing exercises without the app.

P1

Participants reported gaining a range of concrete skills as a result of their app use, including relaxation techniques, greater motivation (demonstrated through commitment to exercise routine), and ability to focus. Generally, app use was discontinued after a skill was acquired; however, the app could be retained in the case that symptoms reemerged to reinstate the desired behavior (see “safety netting” below).

#### Social Connectedness

Three participants reported using an app as a means of enhancing connectivity with individuals and community and engaging with the wider world.

...it’s good to sort of reconnect with like, people you haven’t seen for ages or...people that are going through the same kind of journey as you.

P9

Each of these participants used a different type of app and although these apps may not typically be considered in the context of mental health care (eg, social media and game apps), their role in enabling ongoing social interaction was viewed by participants as a positive influence on mood. This could be through increasing motivation to make new friends and engage with the wider community or through enhancing existing relationships by encouraging reconnection and quality time.

I found that I wasn’t as upset...or depressed, when I was out there playing it [Ingress] and the friends I made through it helped.

P3

...you still sort of keep in contact with what sort of going on in the world. It does make you sort of happy that you still communicate and you're still out there communicating with people.

P9

Like my daughter’s not here tonight, so I probably won’t do it [Smiling Mind] for myself, but when she is, you know, there’s the expectation that, you know, that we’d do it together and that I’d stop [my other activities].

P4

Just it [Ingress] got me out exploring the world around me rather than sitting at home and doing nothing, and moping about. You know, I was out there, I’ve been all over Victoria. I’ve even flown interstate for the game. It has really helped me engage with the world around me.

P3

Although experiences using apps for social connection were generally positive, with an intention to build and foster social relationships, the potential for negative experiences and social conflict was not overlooked.

And then, yeah, sometimes you might get a comment up on Facebook, and someone will take it the wrong way and it'd be–fights all over the place. Yeah, that's where it can sort of turn bad, but nine times out of ten it's usually pretty good.

P9

**Table 1:** Characteristics of the participants.

Characteristics		Participants (N=16), n (%)
**Gender**		
	Male	7 (44)
	Female	9 (56)
**Age in years**		
	<25	3 (19)
	25-44	7 (44)
	45-65	6 (38)
**Employment**		
	Employed	11 (69)
	Unemployed	5 (31)
**Highest level of education**		
	Year 10 or below	2 (13)
	Year 11	1 (6)
	Year 12	1 (6)
	Certificate or diploma	5 (31)
	Bachelor degree or higher	7 (44)
**Depression severity (Patient Health Questionnaire, 9-item)**		
	Minimal or mild (3-9)	3 (19)
	Moderate (10-14)	4 (25)
	Moderately severe (15-19)	8 (50)
	Severe (>20)	1 (6)

**Table 2:** Participation in Target-D and mobile app use for depressive symptoms.

Participant	Depression symptom severity (PHQ-9)^a^	Days since Target-D treatment recommendation	Web-based tools recommended	Apps used, n
P1	Moderately severe	229	No	1
P2	Mild	303	Yes	1
P3	Moderate	178	Yes	1
P4	Moderate	119	No	1
P5	Moderately severe	197	No	2
P6	Moderately severe	319	No	0
P7	Moderately severe	385	No	3
P8	Moderate	369	No	2
P9	Mild	99	Yes	1
P10	Moderately severe	203	Yes	0
P11	Mild	147	No	1
P12	Moderate	420	Yes	0
P13	Moderately severe	184	Yes	0
P14	Moderately severe	354	No	1
P15	Moderately severe	395	No	0
P16	Severe	396	No	1

^a^PHQ-9: Patient Health Questionnaire, 9-item. Level of depression symptom severity is based on PHQ-9 [26] depression scores.

**Table 3:** Mobile apps utilized by participants.

Type of app and app name		Referral source	Used by
**Mindfulness or relaxation**			
	Mindfulness^a^ (3 apps)	Midwife, Psychologist	P1, P5 (2 apps)
	Smiling Minds	General practitioner	P4
	Relax+	Psychiatrist	P14
	Meditation^a^	Self-directed internet search	P16
**Fitness**			
	Exercise^a^	Inbuilt on phone	P2
**Gaming**			
	Ingress	Social network or friends	P3
**Cognitive**			
	SAM App^b^	Self-directed internet search	P7
	NeuroNation	Self-directed internet search	P7
	MindTools	Self-directed internet search	P7
	Psych Me Up	University studies	P8
	Mood Switch	University studies	P8
**Social media**			
	Facebook	Social network or friends	P9
**Workplace well-being**			
	Equipt	Workplace or organization	P11
**No app used**			
	N/A^c^	N/A	P6, P10, P12, P13, P15

^a^Purpose of app described where app name was not known or not specified.

^b^SAM: Self-help for Anxiety Management App.

^c^N/A: not applicable.

#### Inquisitive Trials

Overall, 4 participants trialed at least one app briefly, usually over days to a few weeks. These participants were likely to try more apps with an average of 2 apps each. They expressed curiosity about a variety of different self-management strategies and described actively seeking and downloading apps based on self-directed research, recommendations, and personal interest.

Well, when I was actually at uni, I remember reading about it [Psych Me Up] because I studied psychology. I remember seeing a documentary about it so I downloaded it. It sounded interesting.

P8

I Google it [SAM App]. I Google search it on the *internet*. And, you know, if you will go to the *internet*, [there] is a whole world of people talking to each other...so they recommend to someone and I reading that thing.

P7

However, curiosity alone was insufficient for continuing use. Although these participants generally acknowledged that apps could have a positive impact on depression management, an inability to engage with the app was the most commonly cited reason for discontinuation, though motivation and lack of perceived benefits were also important factors.

Like when I first got it [used] once a day or a something like that for a period of about a week. But then I got over it.

P8

It [mindfulness app] didn’t add anything...I guess it didn’t detract, it didn’t make anything worse, but it didn’t add anything to my armoury, I guess, my tool kit, as keeping myself sane, I suppose, it didn't add.

P5

Personal compatibility was also a key factor commonly associated with a positive attitude toward app use. An individualized approach to depression management was highly regarded because participants often identified personal traits that would hinder or enable the use of certain apps. There was a strong desire for personalized features.

Something that sounds personal to me might be helpful.

P8

I’m sure [it] would be useful for many other people—an app with prompters, but for me to interact with it, I wouldn’t do it regularly.

P5

#### Safety Netting

Two participants reported that they kept an app on their phones that they did not use but retained “just in case.” Both participants had been recommended the app through formal channels (psychiatrist and workplace). One participant had previously used the app successfully to acquire skills; she felt that she no longer required it on a frequent basis but kept it installed to “top up” her skills when she felt her symptoms returning:

...there are afternoons where if I start to fall back into the pattern of not being able to sleep, then I’ll go back to using it. Yeah. So, it’s not an app I would ever delete off my phone, if that makes sense.

P14

Another participant reported that although she had not used her workplace stress management app aside from an initial cursory examination, she intended to do so if she encountered difficult situations resulting in emotional distress. This suggests that an app could be kept as a safety net and used following a precipitating event to guide further management.

...being in the police force, obviously, I attend a lot of critical incidents and that sort of thing, so if there is critical incident that particularly sort of, you know, affecting for me, I might then go into it [Equipt] just to sort of do something about that.

P11

Apart from the potential guidance offered by the app during distressing situations, simply knowing that an app is available for this purpose can provide a sense of comfort.

I just like that it’s there.

P11

### Nonuser Perspectives of Mobile Apps

Five participants had never used an app for their mental health. Perspectives were varied with 3 participants expressing a positive view, one who felt apps were unhelpful, and one who was ambivalent. Among these participants who felt positively toward apps but did not use them, access was the most common barrier. The chaotic nature of the app marketplace and lack of knowledge regarding suitable apps could impede app selection. Additionally, a deficiency in technological competency, perhaps correlating with age, could hinder the use of apps.

...I’d be happy to do it if I knew how to do it [but] I don’t know how to download apps...I need help with technology. Like, I’m 58 and I didn’t grow up in a technological age and so do find that I lack confidence with technology.

P12

...an app could be really useful, especially for the younger generation. For older people, I’m not sure. I don’t really know.

P13

The perceived discordance between technology and mental health treatment was also a reason for nonuse because some participants did not view technology as a natural response to seeking treatment for their mental health. One nonuser reported concerns about excessive dependency on apps, focusing on the possibility of addiction and social disengagement.

It’s not something I kind of...I don’t usually look to technology to fix my issue.

P13

I think we can live vicariously through a screen and that and that I think has some issues in living life in 2.5 inches of screen when there's a whole big horizon out there to look out.

P6

Another viewpoint was that although apps could be beneficial for mental health to an extent, it cannot act as a replacement for social support and human connection. Consistent with the views of many app users, an app can be considered as a supportive tool for depression management but not as a treatment in itself.

I think ultimately though, people who need to know they're cared for by another person, a real person. An app can help but I reckon fundamentally as humans, we want someone to know we’re hurting or struggling, and we need more than an app...but I think it’s a good idea to have both.

P13

A few participants who did not report current app use expressed an inclination to use apps if their situation deteriorated similar to the safety netting behaviors reported by some app users. This suggests that in contrast to the statements above, these participants perceived apps as an effective option for mental health treatment. Their nonuse of apps could be considered in part a reflection of the fact that they did not consider themselves to be currently in need of treatment.

I would go [to an app] when I'm really struggling on a negative downer.

P15

I think if my situation with my mental health got to the point where it prevented me from doing day-to-day things...I think it would be quite beneficial if it came to that point because, once again, it is very accessible.

P10

### Influence of Health Care Providers

Health care providers had a role in facilitating the initiation of mobile app use for depressive symptoms. In this study, 4 app users engaged with an app following a direct recommendation from a health care provider, which included a general practitioner, psychologist, midwife, and psychiatrist. These were all meditation or relaxation-type apps, and participants demonstrated a willingness to try these apps on the basis of professional suggestion and generally persisted with use if they found it helpful.

I use an app on my phone which is—it’s like a meditation app—it was what my psychiatrist recommended. So, yeah, I’ve been very disciplined when he suggested to give it a go and—yeah—and use and give it a go.

P14

However, other app users perceived their app use for mental health independent of professional health care and did not involve their health professionals. There was a tendency among these participants to view professional therapy as separate to their own personal self-directed strategies.

No, I don't think [general practitioner knows about app]...it's my own research.

P7

I don’t think [my general practitioner knows about this app], no, because it is pretty specific.

P11

Regardless of whether or not they were recommended apps, participants demonstrated a sense of trust in guidance from professionals with the perception that these recommendations had a strong basis in experience or knowledge.

I know that my psychiatrist was recommending [Relax+] to other clients, and he found a lot of people found it worked and that was the feedback he got, so that’s why it was an app that he would recommend to people.

P14

I’d rather ask a counsellor or a doctor what they would recommend.

P1

I mean definitely a professional, I’d listen more so than just say on the streets and such...because, you know, you sort of have your belief in the professional industry who have done their hard yards of study and work and, you know, you’d sort of put yourself out there in their hands a bit and they have more knowledge than yourself. And plus, they deal with so many different people in the same field.

P15

The role of health care providers in ongoing app use was unclear because no app users reported involving their health care providers following the initial recommendation.

## Discussion

### Principal Findings

This is the first qualitative study exploring the patterns of mobile app use for depression in a primary care setting, revealing that many participants are already utilizing mobile apps as a way of improving or maintaining their mental health. Prior mental health app research has primarily focused on the prevalence of use and acceptability and attitudes toward apps [12,13,22,30,31] and this, therefore, provides a unique insight into natural app use behavior and the potential role of mobile apps for managing depressive symptoms in the community.

Four main patterns of use were identified, which were not mutually exclusive; some participants indicated different patterns of use of the same app over time. A minority of participants did not report using any app, which is consistent with previous reports of the prevalence of app use in primary care populations [23]. Among those who did report active app use, the most common patterns were using apps to develop or reinforce a particular skill or to enhance social connections.

Importantly, the results of this study suggest that primary care patients use a wide range of apps to manage their depressive symptoms and not only those that are specifically designed for depression management. Interestingly, however, the apps used did encourage engagement in activities that have evidence for efficacy in combating depression, such as acquiring skills through exercise [32] and mindfulness [33] and social connectedness [34]. This suggests that when looking to improve their mental health, primary care patients gravitate naturally toward evidence-based management strategies and should be empowered to engage in techniques they perceive as helpful. This is supported by a study of evidence-based mental health app recommendations suggesting that apps focusing on behavioral activation can empower users to learn beneficial skills and better engage with therapy [35]. Furthermore, self-efficacious behavior changes were demonstrated in a survey where 90% of mental health app users reported increased motivation, desire to set goals, confidence, and intention to be emotionally healthy from their app use [36].

Few app users reported negative effects of apps, although some concerns about addiction were raised by nonusers. That said, the relationship between social networking tools and depression is complex with research suggesting a dual relationship influenced by numerous mediating and aggravating factors. There is a strong implication that the nature of social media use may have more impact on depression symptoms than frequency of use and that the personal perception of Web-based interactions influences mental health; it may be positive if it increases social support or conversely and negative if it increases social comparison and rumination [37]. How patients use and perceive networking tools for social connectedness may be more important than the frequency or type of app used.

Participants did not consider apps as a standalone replacement for traditional professional management but rather as a tool to support and enhance treatment, a viewpoint consistent with clinicians’ beliefs in a qualitative study regarding apps for severe mental health issues [38]. The value in professional mental health treatment was strongly recognized, but participants utilized apps to support activities that coincided with their treatment beliefs, often when accessibility barriers hindered traditional mental health care.

It is also important to acknowledge the role of health care providers in app use for depression. One third of app users (4/11) directly engaged with their app following a health professional’s suggestion compared with 10% of app users in a broader primary care sample in the United States [23]. The higher proportion of health professional recommendations in this study may be a reflection of a number of factors. First, the app marketplace has grown significantly since 2013, as have the popularity and acceptability of apps generally. It is possible that recognition of apps as a genuine option for mental health care has likewise grown. Second, in 2015, a review of Australia’s mental health system resulted in a shift in policy direction toward digital solutions to improving care. As a result, general practitioners have been provided with greater guidance on what mental health apps are available and how to incorporate them into treatment plans [39]. The high level of trust in general practitioners expressed by many participants in this study suggests that they can have a strong influence on initiating app use for depression symptoms. However, it is important to note that the influence of professional support in continuing app use was not determined in this study. This may be due to the perceived discordancy between health care and app use because 70% of respondents in a primary care survey reported that it was not at all or only a little important for physicians to know about their health app use [23]. However, there is some evidence that the therapeutic relationship between care providers and patients has an impact on sustaining app use and enhancing mental health treatment in primary care [22] and this should be an area for future investigation.

In light of the findings above, we suggest that general practitioners consider a holistic approach when supporting patients to engage in mobile technology to improve their mental health. The complexity of depression, which presents with differing physical, psychological, and social issues in every case warrants an approach that is personalized to each individual’s circumstances and needs. A social networking app, for example, may not have evidence for improving depression but for some patients, it may be a more acceptable way of building social connection than more traditional interventions [40]. Thus, considering a person-centered approach and tailoring app recommendations accordingly may provide a more effective approach to integrating apps into depression management. There is some evidence to support this approach, for example, a pilot study of IntelliCare, a modifiable skills-based suite of mental health apps each focusing on a specific task or activity, demonstrated improvements in depression and anxiety scores among app users [41].

It is important to be aware that a wide range of apps, not simply those that are specific to mental health, may be relevant to depression care. Understanding personal preferences and goals may help general practitioners assess what app, if any, would be suitable for an individual and guide appropriate recommendations, especially as the ever-expanding and chaotic nature of the app marketplace remains a significant challenge [14,18]. Ultimately, the end is more important than the means and if patients can view an app as a way of improving or maintaining their mental health, it can reasonably be considered as a part of their mental health care.

It should be noted that, in exploring patterns of app use, one pattern identified was that of nonuse. By employing a personalized approach focusing on treatment goals rather than apps themselves, general practitioners may find that some nonusers can transition into the “inquisitive trial” category as a starting point. This is particularly likely when the main reason for nonuse is lack of knowledge about what app to use or how to use it. However, general practitioners must also remain cognizant that apps will not address unmet needs for all of the 65% of Australians currently not accessing mental health treatment. They may more usefully be construed as one referral option available to general practitioners within a matrix of other options of varying modalities, intensity, and treatment focus.

### Limitations

There are some limitations to this study. First, the findings are based on a small number of general practice patients recruited in Melbourne, Australia, and whether these patterns of use are evident outside this group is unclear. Second, as participants were all under 65 years of age and were recruited from a trial which includes Web-based interventions, this could have led to selection bias because the sample involves a relatively younger and more technology-inclined population. Hence, generalization to all primary care patients would not be feasible.

Further, a substantial proportion of the sample reported depressive symptoms toward the severe end of the spectrum. Guidelines would suggest that this group should be recommended more formal treatment than self-help via an app. However, as a study of the natural use of apps, we see that the inclusion of these participants is a strength rather than a limitation. By understanding patients’ existing management strategies (including apps) and treatment preferences, general practitioners may be better placed to engage patients in more intensive mental health interventions when appropriate, particularly given that apps were viewed as a complement to rather than substitution for formal treatment.

In addition, owing to the cross-sectional nature of this study, the patterns of use identified relate only to the point at which interviews were conducted and do not assess the impact of changes in depressive symptoms over time. We previously investigated use of mental health websites over a 9-year period and found the primary care patients were more likely to report the use of these websites when their depressive symptom severity increased [42]. Given that “safety netters” and some nonusers reported they would consider using an app in the face of worsening mental health, a longitudinal study of whether these intentions are translated into actions would add to our understanding of the patterns of app use.

Finally, this study focused on the perspectives of app users and not the effectiveness of the apps themselves. It is possible that the app use reported here resulted in improved mental health; it is also possible that it made no difference or worsened symptoms. More research is required to explore the potential benefits and harms of app use. For instance, although there is evidence that social media apps may facilitate social connectedness [40], little is known about the effects of dependency on mobile apps in depression. It is important for future research to evaluate the patterns of app use that may exacerbate depression as well as those that facilitate its management.

### Conclusion

The rise of mobile technology heralds a new era in community mental health treatment. There is growing interest from clinicians, consumers, and developers in harnessing the potential for mobile apps to address some of the barriers to traditional mental health care. The results of this study suggest that like any mental health intervention, apps are used only to the extent that they align with patient preferences and treatment goals. Further, although individual apps have limited evidence for effectiveness, patients naturally gravitate toward apps that encourage behaviors known to improve depression. General practitioners may capitalize on this by considering apps as another option in their referral toolbox, a new way for patients to access and engage with old interventions and not a new intervention altogether.

## Appendix

Multimedia Appendix 1 Interview discussion guide.

## Abbreviations

PHQ-2: Patient Health Questionnaire, 2-item
PHQ-9: Patient Health Questionnaire, 9-item
