# Supplementary material for: Mobile App Use by Primary Care Patients to Manage Their Depressive Symptoms: Qualitative Study
Source: J Med Internet Res. 2018 Sep 27;20(9):e10035. doi: 10.2196/10035 (PMC6231897; doi:10.2196/10035)
Supplement: Multimedia Appendix 1 [file jmir_v20i9e10035_app1.pdf]

Appendix A: Interview discussion guide

We are interested in hearing about your experiences and the ways you manage your mental well-being now that you've been involved in this study for a little while. This will take around 10-20 minutes and you can stop at any time. The information you provide will be confidential. Do you have any questions?

Before I go any further, we would like to record this conversation just so we have an accurate record. You can ask me to stop the recording at any time, is this okay?

Once I start recording I'll state your study ID number and ask for your permission to continue.

Participants who agree to recording will be asked to provide recorded verbal consent as follows:

"This is a Target-D interview with participant [ID]. Before we start, can you confirm that you're happy to have this session recorded and that you understand you can ask to stop recording at any time?"

1. Firstly, how have you found being involved in Target-D?
    a. How have you found the communication from us?
    b. How have you found the surveys?

2. Since you've been involved in Target-D, what kinds of things have you done to manage your emotional health/mood?
    a. How did you come across this/these?
    b. How did it help with managing your mood?

If not mentioned in response to Q2:

3. I'm interested in whether you've used any self-help resources to manage your mood? Anything else?

Prompt with 'anything else?' after responses. Proceed with following questions for managing emotional health/mood/symptoms (if not answered prior in responses):

4. Have you read any self-help books?
    a. Which one(s)? How helpful was it /were they?

5. Have you used any internet programs on your computer?
    a. Which one(s)? How helpful was it /were they?

6. Downloaded any mobile apps to your phone?
    a. Can you tell me about this/these?
    b. What is the name of the app?
    c. Is it free to download or does it have a fee?
    d. What was good about it? What was not so good about it?
    e. How useful/helpful was it?

f. Duration of use: When did you start? How long did you use it for? How often do/did you use it?
g. What made you start/stop using it? When would you use it?
h. How did you use it?
i. Where did you use it?
j. How did you come across this/these? Who recommended it?
k. Would your GP know about it?
l. Would you recommend it to others?
m. What would you like to see in future apps?

If no apps mentioned in responses:

a. What are your thoughts about using mobile apps for your mental well-being?
b. Do you think mobile apps would be good or not good for people with depression?
c. What would you like to see in future apps?

7. What has been most useful for managing your mood?

8. Is there anything else you would like to add?

Closing statements:

Would you like a copy of the final report once it is done?

Thank you for your help with this study. We also have a $20 gift voucher to thank you for your time.
